# Collaboration between general dental practitioners and dental hygienists: a qualitative study

**DOI:** 10.1186/s12913-022-07933-3

**Published:** 2022-04-14

**Authors:** Joost C. L. den Boer, Brigitte A. F. M. van Dam, Wil J. M. van der Sanden, Josef J. M. Bruers

**Affiliations:** 1 Department of Oral Public Health, Academic Centre for Dentistry Amsterdam (ACTA), Department of Research & Information, University of Amsterdam and VU University Amsterdam, Royal Dutch Dental Association (KNMT), Amsterdam, Netherlands; 2 Department of Research & Information, Royal Dutch Dental Association (KNMT), Amsterdam, Netherlands; 3 Department of Dentistry—Quality and Safety of Oral Healthcare, Radboud Institute for Health Sciences, Radboud University Medical Centre, Nijmegen, the Netherlands

**Keywords:** Oral health care, Leadership, Goals for collaboration, Esponsibilities, Formalization, General dental practitioners, Dental hygienists, Collaboration, Dentistry, Qualitative study

## Abstract

**Background:**

Influenced by governmental measures, collaboration in oral health care practices in the Netherlands has increased in recent decades. Previous studies on this subject have mainly concerned the composition of the staff or have been normative rather than descriptive. Based on the existing literature, four aspects were expected to be of significant influence on the collaboration on oral health care practices: goals, leadership, the allocation of tasks and responsibilities and formalization.

**Methods:**

The aim of this study was to describe a classification of collaboration between general dental practitioners and dental hygienists within oral health care practices in the Netherlands. Eighteen semi-structured face-to-face interviews were conducted in nine oral health care practices, which differed with regard to both practice characteristics and characteristics of the principal general dental practitioner. In all practices, the principal general dental practitioner and one dental hygienist were consulted. The interviews were conducted in the practices of the respondents and were held between October 2019 and July 2020. The interviews were analyzed through theoretical thematic analysis using Microsoft Word 2010 and Atlas.ti 8. Three researchers coded and analyzed three practices, and discussed their results. Based on their mutual conclusions, one researcher analysed all practices. The final results were reviewed and approved by the other two researchers.

**Results:**

Different factors influencing the collaboration between general dental practitioners and dental hygienist in the Netherlands were found. The most important factors seemed to be leadership style and goals for collaboration. Leadership style varied from very directive to very supportive and seemed to be connected to the allocation of responsibilities. Goals for collaboration varied from predominantly patient-related to mostly practice-related. Formalization appeared to be more present in larger practices and practices that are affiliated to a dental chain.

**Conclusions:**

Based on leadership style and goals for collaboration, a classification was identified. This classification is recommended as a starting point for further research on directive and supportive leadership in oral health care practices.

## Background

In recent decades, oral health care in the Netherlands has been affected by changing laws and regulations aimed at task redistribution and collaboration in teams [1]. The Commission Innovation in Oral Health Care—in Dutch, *Commissie Innovatie Mondzorg* (CIM)—stated in its advice on the future of oral health care in the Netherlands that the guiding principle in collaboration is that various forms of oral health care are delivered by the professional who is best equipped [2]. The arrangement 0f collaboration in oral health care has been classified in several studies. Bruers, den Boer [3] distinguished four types of oral health care practices (OHCPs) based on collaboration between general dental practitioners (GDPs) and practice ownership. Another classification in four types is based on the proportion of dentists in the staff, which can be small or large [4]. A smaller share of GDPs in the combined hours worked by all staff members implies more task delegation. Jerković-Ćosić [5] also took task delegation and differentiation as a starting point for her classification. She recognized six types of OHCPs based on delegation and the reference of tasks to dental hygienists (DHs) and prevention assistants inside or outside the practice.

After consulting experts, Van Dam, Bolk [6] defined collaboration in oral health care as “the cooperative provision of oral health care by one or more GDPs, dental specialists and/or other oral health care professionals, based on consensus and mutual confidence.” This definition focuses more on the health care-related aspects of collaboration. Another common classification of collaboration is in accordance with this definition. It distinguishes three types of collaboration: multidisciplinary, interdisciplinary and transdisciplinary [7, 8]. For these terms, different definitions exist, and the distinction is not always clear [7]. In general, multidisciplinary refers to different disciplines working independently on different aspects of a project. Transdisciplinary indicates working on a common problem in accordance with an agreed conceptual framework but also considering differences. Interdisciplinary collaboration is characterized by a holistic approach in which the specific knowledge of all actors is utilized [9].

Apart from categorizations, several studies have investigated the factors affecting collaboration in general practices and OHCPs. Mickan and Rodger [10] identified 18 factors affecting the effectiveness of health care teams. Seven related to the organizational structure: clear purpose, appropriate culture, specified task, distinct roles, suitable leadership, relevant members and adequate resources. Four related to individual contribution: self-knowledge, trust, commitment and flexibility. Furthermore, seven aspects concerned team processes: coordination, communication, cohesion, decision-making, conflict management, social relationships and performance feedback. In another study, the same authors suggested that an efficient team would benefit from open communication, a focus on the patients’ needs and understanding and experiencing the benefits of working in teams [11]. Wake-Dyster [12] distinguished three factors of interest in the effectiveness of teams: a clear goal, the size of the team and a clear model of leadership. D'Amour, Ferrada-Videla [13] observed five concepts: sharing, partnership, power, interdependency and process. Lemieux-Charles and McGuire [14] concluded that diversity of expertise and involvement in decision-making were the most promising factors for organizational effectiveness. Schaub and Bruers [15] mentioned four aspects that are crucial for the way in which collaboration is shaped: leadership, type of teamwork, goals of collaboration and “the human factor.” Based on a literature review, Reeves and colleagues [16] distinguished five elements of teamwork: shared team identity, clear roles and goals, interdependence, integration and shared responsibility. Xyrichis, Reeves [17] elaborated upon this classification in six aspects: shared commitment, shared identity, clear team goals, clear roles and responsibilities, interdependence between team members and integration between work practices.

In different studies on collaboration, different terminology was used, which might be confusing [18]. However, the classifications overlapped in content. Four main themes can be distinguished: shared goals, leadership, the division of tasks and responsibilities and formalization. The first three of these terms occurred in most of the cited studies; formalization did not. The latter term refers to the standardization of work activities and procedures [19, 20].

In Dutch oral health care, a large variety is expected in the collaboration between GDPs and DHs. This expectation is based on the differences in the authorization of the most common oral health care providers, which are GDPs, DHs and prevention assistants [1]. The authorizations of medical and dental professional groups to perform procedures are described in the Individual Healthcare Professions Act [21]. GDPs are authorized to autonomously carry out a broad range of dental procedures. DHs can autonomously perform a limited number of procedures, some of which they can only perform after a referral by a GDP. Prevention assistants are only allowed to perform procedures under the supervision of an authorized professional. Therefore, in GDP—DH collaboration, both dependency and independency apply. In GDP—GDP collaboration, both actors can act independently, and in collaboration between a GDP or DH and a prevention assistant, the latter is always subordinate.

The aim of this study is to identify a classification of collaboration between GDPs and DHs. To achieve this goal, a qualitative study was carried out aiming to obtain insight into how collaboration in the provision of oral health care is shaped in daily practice in the Netherlands. Special attention was given to the four distinguished themes of collaboration: shared goals, leadership, the division of tasks and responsibilities and formalization. In contrast to previous research on this subject, this study is descriptive rather than normative. Insight into the actual ways in which collaboration is shaped can provide valuable information on the need and opportunities to improve practice and oral health care delivery.

## Methods

For this explorative study, semi-structured face-to-face interviews were conducted. The data from these interviews were analysed through theoretical thematical analysis, as described by Braun and Clarke [22]. Nine OHCPs were selected. To consider both sides, in each practice, both a GDP and a DH were interviewed. All GDPs were the principal GDP: six were practice owners and three were GDP managers. At the start, OHCPs were selected from the Data Stations Network of the Dutch Dental Association [3, 23]. This is a long-running research project mainly concerning various aspects of the dental profession. Initially, convenience sampling was applied, selecting OHCPs varying in size, region of establishment, affiliation with a dental chain and the sex and year of birth of the GDP/practice owner. After interviews in eight OHCPs, data saturation seemed near. However, after inspection of the characteristics of practices involved in the study, it was noticed that ‘small’ OHCPs were underrepresented in the sample. As the size of the practice can be a factor in collaboration, it was decided to additionally include a genuinely small OHCP—with one GDP and one DH. A brief overview of the characteristics of the participating practices is shown in Table 1. These characteristics were partly registered in the administration of the Data Stations Project and partly obtained from the interviews.

**Table 1:** Characteristics of the oral health care practices involved in the study

***Practice***	1	2^a^	3	4	5	6	7	8	9
*Number of general dental practitioners (GDPs)*	4	n/a	9	16	15	7	4	6	1
*Number of dental hygienists (DHs)*	2	n/a	3	3	5	3	4	3	1
*Number of treatment units*	4	n/a	8	13	16	8	5	8	2
*One or more prevention assistant*	yes	yes	yes	no	yes	yes	yes	yes	no
*Number of active patients*	11,000	75,000	10,000	25,000	17,000	9,250	4,250	8,000	1,800
*Year of establishment*	2012	2010	2008	1994	2010	1988	2008	1989	1998
*Year of introduction of DH*	2012	2010	2008	2001	2010	1992	2008	1995	2016
*Affiliated with a dental chain*	no	yes	no	no	yes	yes	no	yes	no
*Region*	west	west/south	west	west	east	west	west	south	west

^a^The GDP is the founder and owner of a chain of 10 practices in the west and south of the Netherlands. In total, these practices employ approximately 450 persons and serve 75,000 patients. Both GDPs and DHs work in several practices within this dental chain, and both provided information that applied to the dental chain in general rather than to a specific practice

*n/a* not available

All interviews were conducted by JdB between October 2019 and July 2020 in the OHCPs in which the respondents worked. Visiting the practice has several advantages. It minimizes the efforts of the respondents to participate, and it offers the interviewer insight into the work environment that is discussed.

GDPs and DHs were interviewed separately to provide confidentiality to talk about the positive and negative aspects of their collaboration. This applied especially for DHs, as on all occasions, they were employees and the GDPs were owners or managers of the practice. A member check was performed: all respondents were sent a summary of the interview—written by JdB—and given the opportunity to indicate whether the interviewers’ summary matched their perceptions.

The analyses were performed using Microsoft Word 2010 and Atlas.ti, version 8. To this end, all interviews were audio-recorded and transcribed verbatim by an independent transcriber. The theoretical thematic analysis was performed following the five phases -with reporting being the sixth phase—as described by Braun and Clarke [22].

In order to obtain familiarization with the data (phase 1), JdB anonymized the transcripts and drafted summaries for member checks. All researchers did the initial reading per practice; they selected an OHCP and read both interviews before moving on to another practice. This helped to assess whether views of the GDP and the DH on their practice correspond.

In phase 2, initial codes were assigned manually. After that, synonyms and near-synonyms—for instance, “DP has final responsibility” and “GDP is ultimately responsible”—were combined. The search for themes (phase 3), was performed manually and individually by each researcher.

For the review of the themes (phase 4), the results of all researchers (BvD, JB and JdB) regarding Practice 3 were discussed in a three-way conversation in which the coding framework was developed. This framework was applied in the analyses of two more practices (4 and 7) by all three aforementioned researchers. After this step, the coding framework was discussed further in another three-way conversation. Although it was substantiated that two initial themes—‘leadership’ and ‘allocation of tasks and responsibilities’—were interconnected, the differences between the two themes were too large to merge them together. Furthermore, ‘factors that promote or demote collaboration’ was also considered a theme. Many respondents mentioned these preconditions during the interview, it was considered useful background information that should not be ignored. For practical reasons ‘practice characteristics’ was also established as a theme.

Subsequently, the remaining practices were analysed by JdB according to the agreed coding framework, which is presented in Table 2. The final findings were discussed in a three-way meeting, in which the final description of the themes was established (phase 5).

**Table 2:** Coding framework

Practice characteristics	General characteristics: - Number of owners - Number of employees - Number of patients - Number of dental units - Region of establishment - Affiliation with dental chain - Year of establishment of the practice - Characteristics of the patient population Characteristics regarding collaboration: - Practice layout (designed to promote collaboration) - Year of introduction of DH - Type of employment (self-employed or contract employee) - Presence of other care providers and degree of task reallocation
Goals of collaboration	Goals at patient level: - Reasons for referral or delegation - Rationale behind involvement of specific oral health care provider (profession) - Rationale behind involvement of specific oral health care provider (person) Goals at practice level: - Rationale behind type of practice - Rationale behind (type of) collaboration - Expected benefits of collaboration for the patient population - Expected benefits of collaboration for the oral health care providers - Expected benefits of collaboration for the practice
Leadership	- Vision of the GDP on oral health care - Vision of the GDP on leadership and practice management - Strategy to promote vision among employees - Hierarchy of power in the practice organization - Hierarchy of power in the process of delivering oral health care
Allocation of tasks and responsibilities	- Who does what? - Rationale behind involvement of specific oral health care provider (profession) - Rationale behind involvement of specific oral health care provider (person) - Freedom of employees to make decisions - Responsibility for the results of a specific procedure - Responsibility for the whole treatment/oral health of the patient - Freedom for different oral health care providers to address one another
Formalization	- Systems and procedures for the modelling and preservation of collaboration (e.g., meetings and protocols) - Type of employment (self-employed or contract employee) - Ways in which agreements are reached - Compliance with agreements
Factors that promote or demote collaboration	- Factors that promote or demote collaboration in general - Factors that promote or demote collaboration in the specific practice
Other	- Subjects that are not connected to collaboration

The results presented are exemplified with quotes of participants. These quotes are translated from Dutch; an overview of all quotes—in English and Dutch—is presented in Table 3. After the identification of the aspects affecting collaboration between GDPs and DHs, a classification was devised using these characteristics. After that, the nine participating practices were classified based on the statements of both GDPs and DHs regarding these aspects. Three practices were labelled by BvD, JB and JdB independently. The researchers discussed their findings and came to consensus. The six remaining practices were labelled by JdB in line with the classification of the first three practices.

**Table 3:** Quotes of participating GDPs and DHs, translation in English and original quote in Dutch

quote	English		Dutch
1	… but GDPs in this practice in general are very periodontology minded, hence they consider prevention very important; more important than other things		*... maar de tandartsen hier zijn over het algemeen heel parodontologie minded, dus die vinden de preventie eigenlijk belangrijker dan andere zaken*
2	… seven days a week, fourteen hours a day attention, hospitality		*... zeven dagen per week, veertien uur per dag aandacht, hospitality, gastvrijheid*
3	… investing very much time in patients. Not only in treatment, but also in the person		*... zeer veel tijd aan een patiënt te besteden. Niet alleen aan de behandeling maar ook aan de mens zelf*
4	It is faster, more efficient, etcetera		*Het is sneller, efficiënter, etcetera*
5	So, when a patient comes to you, then you can easily look up who referred this patient to me and why		*Dus als patiënt naar jou komt, dan kun je gewoon kijken wat de reden is en wie die patiënt naar jou heeft verwezen*
6	There’s one treatment plan, one patient file		*D’r is één patiëntbehandelplan en behandelkaart*
7	If an assistant believes a GDP omits something, […] this should come up		*Als een assistente denkt dat een tandarts iets niet doet, […] dan moet dat wel naar boven komen*
8	…team leaders monitor it all. If they know something is not in accordance with our protocol or working methods, they point that out		*De teamleiders houden het al wel allemaal in de gaten. Als zij weten dat het een beetje niet-conform onze protocol of onze werkwijze is, dan wijzen zij er wel op*
9	I am not an advocate of task delegation in the sense that the dental hygienist can drill on their own. Because at the end of the day I get called when a patient gets pain at night. Then I don’t know what treatment was given, but I am responsible for it		*Ik ben geen voorstander van taakdelegatie in de zin van dat de mondhygiënist op eigen houtje gaatjes mag boren. Want uiteindelijk word ik ‘s avonds gebeld als er pijnklachten zijn. Dan weet ik niet wat er gebeurd is, maar ik ben er wel verantwoordelijk voor*
10	… I believe it is very important that dental hygienists, who have mastered the periodontal part of their jobs, should put their effort into that part of the patient treatment. In my opinion, it is impossible to divide time between preventive and curative activities adequately		*… ik vind het superbelangrijk dat die mondhygiënistes, die het parodontologie deel goed beheersen, dáár hun energie in steken voor de patiëntenbehandeling. Het werkt volgens mij niet als zij hun tijd moeten verdelen tussen preventie en curatief*
11	Well, I don’t feel the need right now. Because I am particularly engaged with periodontology, and that’s my passion		*Nou, ik heb nu de behoefte niet, omdat ik me met name bezig houd met de parodontologie. Dat is ook mijn passie*
12	GDPs do want to delegate tasks […] preferably to a dental hygienist. But this requires a multiplication of training positions by three		*Tandartsen willen wel dat er gedelegeerd kan worden, […] het liefst naar een mondhygiëniste. Maar dan moet je daar ook drie keer zoveel van opleiden*
13	Maybe that person has a fresh look, maybe that person can do more for a patient than I can		*Misschien heeft die weer een frisse blik, misschien kan die toch weer nét iets anders voor je betekenen dan ik*
14	If all is according to plan, there are ring binders with all protocols on every location		*Volgens mij zijn, als het goed is, op elke locatie wel klappers aanwezig waar de protocollen in staan*
15		With interns, we even practice, […] I, for instance, sometimes perform the role of patient to enable someone to practice how to accompany a patient from the waiting area	*Met stagiaires wordt zelfs geoefend, […] het ophalen van een patiënt wordt, bijvoorbeeld, van tevoren met mij geoefend, ik speel een nep-patiënt*
16		Yes, things go in accordance with the rules in this practice	*Ja, het is wat schoolser hier*
17		At some point in time, deterioration occurs and then you have to find an alternative way	*Op een gegeven moment komt daar de sleet in en dan moet je weer een nieuwe manier vinden*
18		Basically, the team is too small for that	*Daar is het team gewoon te klein voor*
19		… very few dental hygienists want to work in paid employment	*…er zijn heel weinig mondhygiënistes die in loondienst willen werken*
20		If there suddenly were four time as many dental hygienists, we could have equal negotiations. Because currently, they can demand anything they want	*Maar als er morgen in één keer vier keer zo veel mondhygiënisten zijn, dan kunnen we tenminste ook weer volwaardig in gesprek. Want nu kunnen zij eisen wat ze willen*

## Results

### Goals of collaboration

In all OHCPs, a GDP had taken the initiative for collaboration with one or more DHs. Accordingly, both GDPs and DHs described the goals of collaboration from a GDP’s point of view. Furthermore, the objective for collaboration was defined in two ways: (1) narrowly, as the aims for the treatment of individual patients, and (2) more broadly, as the objectives for the patient population and practice.

With regard to aims at the individual treatment level, patients were categorized into risk groups based on periodontal condition. For this purpose, the Dutch periodontal screening index, the so-called perio-protocol, was used (Van der Velden, 2009). Although this categorization is standardized, there were differences between practices. In practices 6 and 8, the perio-protocol was strictly followed; in Practice 9, the most flexibility occurred, as care providers were allowed to deviate from the protocol if they considered the treatment too costly in relation to the expected benefits.

More variation was found regarding goals for the practice and patient population. Naturally, all GDPs brought up the oral health of their patients as an important reason for collaboration. In Practice 9, the oral health status of the patients was virtually the only reason mentioned. In Practice 3, other aspects—mainly regarding the process of oral health care delivery—were named but explicitly as secondary goals.

“(1)… but GDPs in this practice in general are very periodontology minded, hence they consider prevention very important; more important than other things.”

DH, Practice 3

In practices 1, 2, 4, 6, 7 and 8, other patient-related reasons—such as convenience, comfort and costs—were identified.

“(2)… seven days a week, fourteen hours a day attention, hospitality.”

GDP/owner of small dental chain, Practice 2

“(3)… investing very much time in patients. Not only in treatment, but also in the person.”

GDP/practice owner, Practice 7

In the main part of the practices, the benefits for the process of oral health care delivery were also acknowledged. These benefits include efficiency (practices 1,2 and 3), better opportunities to share patient information (practices 3, 4, 5 and 8) and the opportunity to oversee the entire treatment (practices 4, 5, 6, 7 and 8).

“(4) It is faster, more efficient, etcetera.”

GDP/owner of small dental chain, Practice 2

“(5) So, when a patient comes to you, then you can easily look up who referred this patient to me and why.”

DH, Practice 4

“(6) There’s one treatment plan, one patient file.”

GDP/manager, Practice 5

### Leadership

All practices had a management hierarchy with the principal GDPs at the top. In the process of delivering oral health care, less strict hierarchies were found. Furthermore, the practices differed with regard to chairside hierarchy. In Practice 3, maximum equality was pursued; in all other practices, at least some level of hierarchy was sustained. In most practices, this meant that GDPs had the most freedom to operate and were given the ultimate responsibility for the patients’ treatment. But there were exceptions. In Practice 7, for instance, a very experienced DH was ranked higher in the hierarchy than an inexperienced GDP.

With regard to the style of leadership of the principal GDP, the differences were significant. Some leaders were facilitating and stimulating. The owner of Practice 2, for instance, permitted the dental team to have much freedom in the elaboration of his ideas. Furthermore, the owner of Practice 7 tried to provide a stimulating environment for all staff. The owner of Practice 3 encouraged all employees to share their opinions, solicited and unsolicited:

“(7) If an assistant believes a GDP omitted something, […] this should come up.”

Other principal GDPs adopted a more directive leadership style, which was most apparent in the GDP of Practice 4. When not present in the practice, team leaders (mainly the practice manager) were supervising for him:

“(8)…team leaders monitor it all. If they know something is not in accordance with our protocol or working methods, they point that out.”

GDP/practice owner, Practice 4

In terms of leadership style, the GDP/managers of practices that were affiliated with a dental chain were in between the extremes. They clearly were in charge but engaged employees in decisions.

### Allocation of tasks and responsibilities

Prevention and periodontology were the main focus areas of DHs; GDPs primarily focused on diagnostics and curative treatment. If applicable, prevention assistants performed basic prevention treatments, such as supragingival plaque and calculus removal and oral health care education. The presence of prevention assistants allowed DHs to focus on periodontology and complex prevention, such as subgingival plaque and calculus removal and the treatment of medically compromised patients. In general, GDPs did not utilize all possibilities Dutch law and regulations offer to expand the range of tasks of DHs. For this underutilization, three reasons were mentioned most frequently. Firstly, GDPs—and several DHs—expressed reservations about the extent of task reallocation.

*“(9) I am not an advocate of task delegation in the sense that the dental hygienist can drill on their own. Because at the end of the day I get called when a patient gets pain at night. Then I don’t know what treatment was given, but I am responsible for it.”*

GDP/practice owner, Practice 9

*“(10) ... I believe it is very important that dental hygienists, who have mastered the periodontal part of their jobs, should put their effort into that part of the patient treatment. In my opinion, it is impossible to divide time between preventive and curative activities adequately..”*

GDP/practice owner, Practice 3

“(11) Well, I don’t feel the need right now. Because I am particularly engaged with periodontology, and that’s my passion.”

DH, Practice 3

Secondly, DHs worked without chairside assistants, and some procedures cannot be optimally performed without those assistants. And thirdly, the number of DHs was hardly sufficient to cope with the existent workload:

“(12) GDPs do want to delegate tasks […] preferably to a dental hygienist. But this requires a multiplication of training positions by three.”

GDP/manager, Practice 8

In almost all practices, the continuity of the treatment relationship was important. In Practice 3, however, this relationship was sometimes sacrificed to optimize treatment. A DH explained that her personality and working method did not fit all patients. If this is the case, a patient is likely to benefit from a change of DH:

“(13) Maybe that person has a fresh look, maybe that person can do more for a patient than I can.”

DH, Practice 3

The professional autonomy of DHs varied between practices. In Practice 4, on the one hand, the autonomy was limited; the range of tasks was limited to basic prevention and the freedom to operate was restricted. Therefore, the responsibility of DHs was limited to the correct performance of the tasks commissioned by the GDP. In practices 3 and 7, on the other hand, joint responsibility was established, although the principal GDPs did believe that, as practice owners, they had the final responsibility for all patients.

### Formalization

The use of protocols was very common. Only in the smallest practice [9] was the use of protocols not mentioned. However, practices varied in the ways in which protocols were integrated into daily practice. In practices 1 and 2, protocols were available for all staff, but there seemed to be no policy to stimulate or verify their use. The DH of Practice 2, for example, was not sure whether all locations had a printed version of the protocols.

“(14) If all is according to plan, there are ring binders with all protocols on every location.”

DH, Practice 3

Conversely, in practices 3, 4 and 7, the use of protocols was promoted and monitored. In Practice 3, protocols were incorporated into the dental software; in Practice 4, the use of protocols was monitored; and in Practice 7, the use of protocols was embedded in the onboarding of new employees.

“(15) With interns, we even practice, […] I, for instance, sometimes perform the role of patient to enable someone to practice how to accompany a patient from the waiting area.”

GDP/practice owner, Practice 7

Practices 5, 6 and 8, all were affiliated with a dental chain; that made those practices’ employees obligated to work according to protocols that adhered to clinical guidelines. The parent company gathered data from practices and monitored whether the care delivered was in line with these guidelines. The use of protocols seemed mandatory in practices that belong to this dental chain:

“(16) Yes, things go in accordance with the rules in this practice.”

DH, Practice 6

Another aspect of formalization concerns consultation. Generally, oral health care providers appreciated informal consultation more than formal meetings. Preferably, they consulted a colleague during or immediately after a patient’s visit. Three practice interiors (3, 7 and 9) were even designed to make chairside consultation possible. But even in the best circumstances, direct face-to-face consultation was not always possible due to different work schedules. Therefore, information was also communicated via notes in the digital patient records (practices 2, 3, 4, 5, 6, 7, 8 and 9), email (practices 1 and 2) and internal messages (practices 4 and 7).

Although informal consultation was preferred, several practices (2,3,4, 5 and 7) did have some meeting structures. In all cases, intra-professional meetings were scheduled more frequently than interprofessional meetings. Moreover, it appeared to be quite challenging to preserve the meeting structure. Maintenance is important, and sometimes, a change of approach is necessary:

”(17) At some point in time, deterioration occurs and then you have to find an alternative way.”

GDP/practice owner, Practice 3

In some practices, the maintenance had not succeeded: in Practice 1, the meeting structure had collapsed, and a DH in Practice 6 only remembered one meeting since her introduction into the practice a year before the interview. In Practice 8, informal consultation was scheduled at the end of every day, whereby a clear structure was established. Practice 9 only employed three persons: the two respondents and one dental assistant. All three had exactly the same work schedule. In this practice, the GDP and DH consulted each other in between patients and during lunch breaks. The GDP/owner felt no need for regular consultation time:

“(18) Basically, the team is too small for that.”

GDP/practice owner, Practice 9

Most GDPs prefer to have staff members in paid employment, as they believe self-employment offers them incentives to engage in overtreatment. However, the shortage of GDPs and DHs forces them to adapt to the preferences of the staff members. As a result, DHs—and GDPs—typically are self-employed:

“(19) … very few dental hygienists want to work in paid employment.”

DH, Practice 6

“(20) If there suddenly were four time as many dental hygienists, we could have equal negotiations. Because currently, they can demand anything they want.”

GDP/manager, Practice 8

### Conditions for collaboration

In addition to the four characteristics of collaboration that were distinguished based on the existing literature, all respondents mentioned facilitating factors and barriers for the collaboration between GDPs and DHs: patient preferences and knowledge, the shortage of DHs, the fact that collaboration requires investment and maintenance, personality differences and the need to achieve and maintain a balanced composition of the team. These fact0rs can all affect collaboration.

### A classification of collaboration

With regard to a classification of collaboration between GDPs and DHs, this study provides more insight. For example, the interconnectedness of the factors described should be taken into account. Foremost, leadership and the allocation of responsibilities seem to be interconnected; incidentally, differences with regard to the allocation of tasks seemed to be limited by the shortage of DHs. Principal GDPs with a supportive and stimulating style of leadership seemed more inclined to allocate responsibilities to employees. By contrast, a directive style of leadership appeared to be connected to a limited allocation of responsibilities. In this study, the most apparent example of supportive leadership style was in Practice 3, which was also the practice with the most shared responsibilities. The most directive leader was the GDP/owner of Practice 4, who did not grant DHs much autonomy. There seems to be a connection between leadership and goals for collaboration as well. However, this connection concerns not so much the goals themselves as the degree to which these goals were articulated. On a substantive level, there seemed to be a division into patient-related goals, such as oral health status and comfort, and practice-related goals, such as efficiency. All GDPs and DHs described both patient-related goals and practice-related goals. Based on the frequency in which goals were mentioned and the comprehensiveness of the description, practices were positioned on a scale ranging from “very patient related” to “very practice related.” Practice 9 was the most patient related due to a virtual absence of practice-related goals. Practice 1 trended the most toward practice-related goals.

The differences between practices with regard to formalization seem mainly attributed to size differences. In larger practices and practices that were affiliated with a large dental chain, there appeared to be a greater need for standardization of processes. The exception was the small dental chain, which is called Practice 2 in this study. In this dental chain, practices seemed to be operating independently from each other to some extent, within the vision of the GDP/owner.

**Fig. 1:**
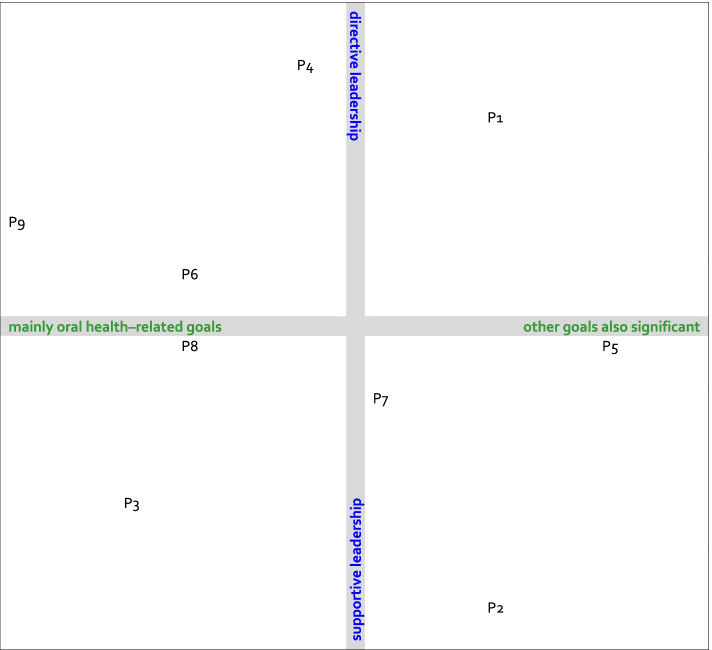
Placement of included practices in the identified classification of collaboration between GDPs and DHs based on leadership style and goals for collaboration. *P* = Practice

All in all, the identified classification of collaboration between GDPs and DHs includes two major factors: goals and leadership. This classification is shown in Fig. 1. The practices were ranked from mainly oral health orientated to a significant orientation to other goals as well and from supportive to directive leadership. The rationale for this ranking is shown in Table 4. Figure 1 shows that the nine practices of this study were dispersed across all quadrants. Supportive leadership seemed to be more related to patient-related goals, and directive leadership was more associated with practice-related goals.

**Table 4:** Ranking and consideration with respect to goals for collaboration and to leadership

Practice	Goals for collaboration	Practice	Leadership
9	Both the GDP/owner and DH mainly presented oral health–related arguments for their professional choices. The GDP brought up the costs for patients but only to emphasize the ability to foster the oral health status of financially needy patients	**2**	The GDP/owner had defined a view on oral health care delivery but allowed the dental teams of the affiliated practices great freedom to shape the collaboration within their team
3	The GDP/owner and DH both valued the oral health status of their patients as the most important factor in their collective efforts, and both overlooked other patient factors. The GDP mentioned aspects regarding the process of oral health care delivery but primarily as a contribution to oral health status	**3**	The GDP/0wner claimed to create possibilities for all professionals to deliver care as well as they can. However, he seemed to monitor DHs to some extent
8	With regard to collaboration, the oral health status of patients was the most important factor, but the GDP/manager did not overlook the financial position of the practice	**7**	All professionals were offered the chance to develop. This requires the freedom to act and supervision. Therefore, leadership in this practice was both supportive and directive. Monitoring, however, was mainly targeted at facilitating the development of the staff
6	With regard to collaboration, the oral health status of patients was the most important factor, but the GDP/manager also mentioned advantages for his own job satisfaction and the financial position of the practice	**8**	The GDP/manager valued and facilitated the development of professionals. In daily practice, he monitored the work of his subordinates
4	The protocols in this practice were very strict. This contributes to an efficient process of delivering oral health care but limits the possibilities to adapt to the patients’ specific needs	**5**	The GDP/manager showed supportive and directive characteristics, which seemed to be quite in balance
7	In the delivery of oral health care, the preferences of patients and their financial restrictions were considered to offer everyone the best oral health care they could afford. Alternatively, a specific goal of the GDPs/owners was to create a learning environment for young professionals and students	**6**	The GDPs/managers leaned toward a directive style of leadership but also showed supportive characteristics
1	A major reason to hire DHs was to keep patients in the practice, which implies the fear of losing patients. Besides that, the practice owner mentioned prevention as a reason	**9**	The GDP/owner generally expressed a directive leadership style. However, he seemed to make an exception for the current staff, as he valued their experience, skills and knowledge very highly
2	The GDPs/owners’ focus was very much on convenience and the well-being of patients, of which oral health status is a part. Moreover, the owner believed efficiency is important to patients but recognized the advantage of collaboration for the practice as well	**1**	One of the reasons to hire a DH was the GDP/owner’s wish to monitor the work of DHs, which was not possible when working with DHs in other practices. In daily practice, the leadership style of the GDP/owner also was supportive to some extent
5	The main reason for the expansion of the practice was the desire to make processes more efficient; good oral health seemed to be taken as a given	**4**	The protocols in this practice were very strict, and the use of these protocols was monitored by the GDP/owner and team leaders

## Discussion

The aim of this study was to identify a classification of collaboration between GDPs and DHs within dental practices in the Netherlands. After 18 interviews in nine practices, a classification was identified based on two aspects: leadership and goals. The latter appears to be closely correlated with the allocation of responsibilities, which is in accordance with the findings of Głód [24]. This interrelationship seems to fit well into the classification into transactional and transformational leadership, which is based on the work of Burns [25] and Bass [26]. This is the most frequently cited classification of leadership studies in health care [27]. The distinction between supportive and directive leadership fits within the situational approach to leadership [28].

In this study, GDPs stressed the importance of recruiting employees who fit with their working methods and views on oral health care provision. They did not mention many efforts to engage employees in these working methods and views. Conceivably, GDPs do not appreciate the possibility of affecting their employees’ behaviour, as they are mainly care providers and consider managing tasks to be of subordinate importance. This is reflected in the fact that clinical subjects are the main focus in dental school and a continuing focus of professional dental education [29–32]. Moreover, the excessive clinical workload may also be a barrier to taking on a leadership role. Elliott, Begley [33] have indicated that both the lack of time and lack of education can be barriers for advanced practitioner nurses to take roles as leaders.

The allocation of tasks mainly depended on the composition of the staff. In practices that employed care providers with less authority than DHs, the latter focused on complex treatments and medically compromised patients. GDPs preferred to delegate patients to DHs for simple preventive treatments as well, but they did not consider this feasible due to a shortage of DHs. Therefore, it seems that the shortage of DHs contributes to the emergence of prevention assistants. In recent years, the Dutch government has tried to address the shortage of GDPs by promoting task reallocation [1]. This study suggests some barriers. Firstly, GDPs oppose wide-ranging reallocation, which confirms previous findings [5, 34, 35]. Secondly, some GDPs have experienced difficulties recruiting enough DHs to meet the amount of preventive work.

In this study, collaboration between GDPs and DHs seemed more multidisciplinary than interdisciplinary, as roles were clearly demarcated. This seems to be in contrast to the fact that dental schools in the Netherlands focus on interdisciplinary education [36]. A possible explanation is that the GDPs in this study all graduated at least nine years prior to the interviews. This was due to the choice to include only practice owners and GDPs/managers—who were all former practice owners—in the study, as graduating GDPs typically start as employees [30].

The fact that big practices and practices that are part of a bigger organization trended toward more formalization is in line with previous research. Dobson and Perepelkin (2011) suggested that pharmaceutical chains standardize processes to minimize uncertainty and the chance of error, and O’Selmo (2018) found that corporate dental practices frequently use protocols and uniform procedures.

The selection of participants in this study was aimed at maximum variety in practices. For this purpose, one ‘one-GDP’ practice was approached to complete the sample. This seems a limited number, as in 2019, approximately two-fifths of OHCPs in the Netherlands were so-called one-GDP practices [1]. However, the employment of DHs in solo practices is limited [37]. Therefore, the inclusion of one solo practice in the study seemed sufficient. A limitation of the selection was that only one of the two major dental chains in the Netherlands was included. Initially, it was planned to include at least one practice affiliated with each chain. However, it was not possible to arrange interviews within a reasonable term due to COVID-19 measures. Although the inclusion of both major chains was desired, the absence of one was not considered an insurmountable problem. After all, the practices affiliated with the excluded chain maintain their names and independent signatures, comparable to the small dental chain that was included as practice 2.

The study has uncovered considerable differences in collaboration. Due to the qualitative research methods applied, this study does not provide information on the prevalence of the characteristics of collaboration in dental practices in the Netherlands. For that purpose, quantitative research methods are preferred. This study suggests that leadership and goals of collaboration are adequate points of focus for future research on collaboration between GDPs and DHs.

## Conclusion

The major factors in collaboration between GDPs and DHs in the Netherlands seem to be leadership style and goals for collaboration. A classification based on these main factors was identified. This classification is recommended as a starting point for further quantitative research on directive and supportive leadership in OHCPs.

## Data Availability

Anonymized transcripts of all interviews are not available online, but are obtainable from the corresponding author on reasonable request. Please note that these transcripts are in Dutch.

## Abbreviations

ACTA: Academic Centre for Dentistry Amsterdam
BvD: Brigitte van Dam
CIM: Commission Innovation in Oral Health Care
COVID-19: Coronavirus Disease 2019
DH: Dental Hygienist
GDP: General Dental Practitioner
JB: Josef Bruers
JdB: Joost den Boer
KNMT: Royal Dutch Dental Association
n/a: Not available
OHCP: Oral Health Care Practice
WvdS: Wil van der Sanden
